# Lessons from an elderly patient with pulmonary embolism caused by protein S deficiency: a case report

**DOI:** 10.1186/s13256-024-04396-4

**Published:** 2024-02-28

**Authors:** Liu Qiang, Li Hong, Shen Min, Wang Hongping, Chen Xian, Li Tianlang

**Affiliations:** 1 Department of Gerontology, The Second Affiliated Hospital, Zhejiang University School of Medicine, Hangzhou, 310002 China; 2 Department of Cardiology, Affiliated Hangzhou First People’s Hospital, Zhejiang University School of Medicine, Hangzhou, 310006 China

**Keywords:** Thrombosis, Pulmonary embolism, Case report, PROS1, Mutation

## Abstract

**Background:**

Lower limb deep vein thrombosis (DVT) concurrent with pulmonary embolism (PE) is perilous, particularly in the elderly, exhibiting heterogeneity with thrombophilia mutations. Tailored treatment is essential, yet sudden deaths complicate causative factor elucidation. This report emphasizes genetic testing necessity in PE patients with thrombophilia indicators, facilitating cause identification, personalized treatment guidance, and family education.

**Case presentation:**

This study details a 75-year-old Chinese woman with DVT and PE, where genetic testing identified thrombophilia, guiding personalized treatment decisions.

**Results:**

Upon admission, the patient, after over 10 days of bed rest, presented chest tightness, shortness of breath, and unilateral leg swelling. Diagnostic measures revealed DVT and a substantial PE. Genetic testing identified a *PROS1* gene C200A>C mutation, reducing protein S activity. Following 2 weeks of anticoagulation and inferior vena cava filter insertion, the patient, discharged, initiated lifelong anticoagulant therapy. A 1-year follow-up showed no recurrent thrombotic events. Family members carrying the mutation received informed and educational interventions.

**Conclusion:**

Genetic testing for thrombophilic predisposition post-PE is crucial, elucidating etiology, guiding individualized treatment, and playing a pivotal role in family education.

## Introduction

Diverging from conventional perceptions, the incidence of deep vein thrombosis (DVT) complicated by pulmonary embolism (PE) is more prevalent than previously acknowledged. In the year 2020 alone, the United States documented 49,243 fatalities attributed to PE [1]. Given the potential interplay of diverse factors, including vascular endothelium, coagulation, and anticoagulation, PE manifests as a markedly heterogeneous condition [2]. Consequently, the approach to PE management necessitates a heightened focus on individualization.

As per the prevailing consensus, individuals with PE harboring underlying causes that are irremovable are recommended to undergo lifelong anticoagulation therapy. Conditions such as protein S deficiency, resulting from genetic mutations, are encompassed within this category of causative factors. Employing genetic testing stands out as an effective modality to pinpoint patients falling into the aforementioned category. However, the current challenge is two-fold: on one facet, the accessibility of genetic testing is on the rise; conversely, the comparative value of genetic diagnosis for PE, particularly concerning tumor-related genetic diagnoses, lacks a clearly defined and timely consensus [3]. Consequently, this case report serves the purpose of sharing our treatment paradigm, which we recognize as somewhat tentative, to conscientize healthcare professionals regarding the criticality of prompt genetic testing for PE patients exhibiting indications of thrombophilia. This testing assumes paramount significance in furnishing fully personalized treatment for individuals afflicted with PE.

## Case presentation

A 75-year-old Chinese female presented with a 2-week history of chest tightness, palpitations, and left lower limb edema. Two weeks prior, she sought care at a community clinic following the onset of palpitations. The electrocardiogram revealed sinus tachycardia with a ventricular rate of 118 beats per minute. The community physician initiated treatment with metoprolol sustained-release tablets (47.5 mg twice daily). Despite a subsequent decrease in heart rate to 56 beats per minute 2 days later, the patient persisted with symptoms, including palpitations, chest tightness, exacerbated symptoms with activity, and pitting edema in the left lower limb.

Upon arrival at our outpatient department, the patient’s pro-BNP was measured at 1760 pg/mL, accompanied by evident dyspnea on exertion, leading to the decision for hospital admission. The patient had no prior history of cardiovascular disease, diabetes, or tumors. However, a recent bout of knee joint pain had rendered the patient bedridden for more than 10 days 1 month earlier. There was no familial history of similar ailments.

**Fig. 1:**
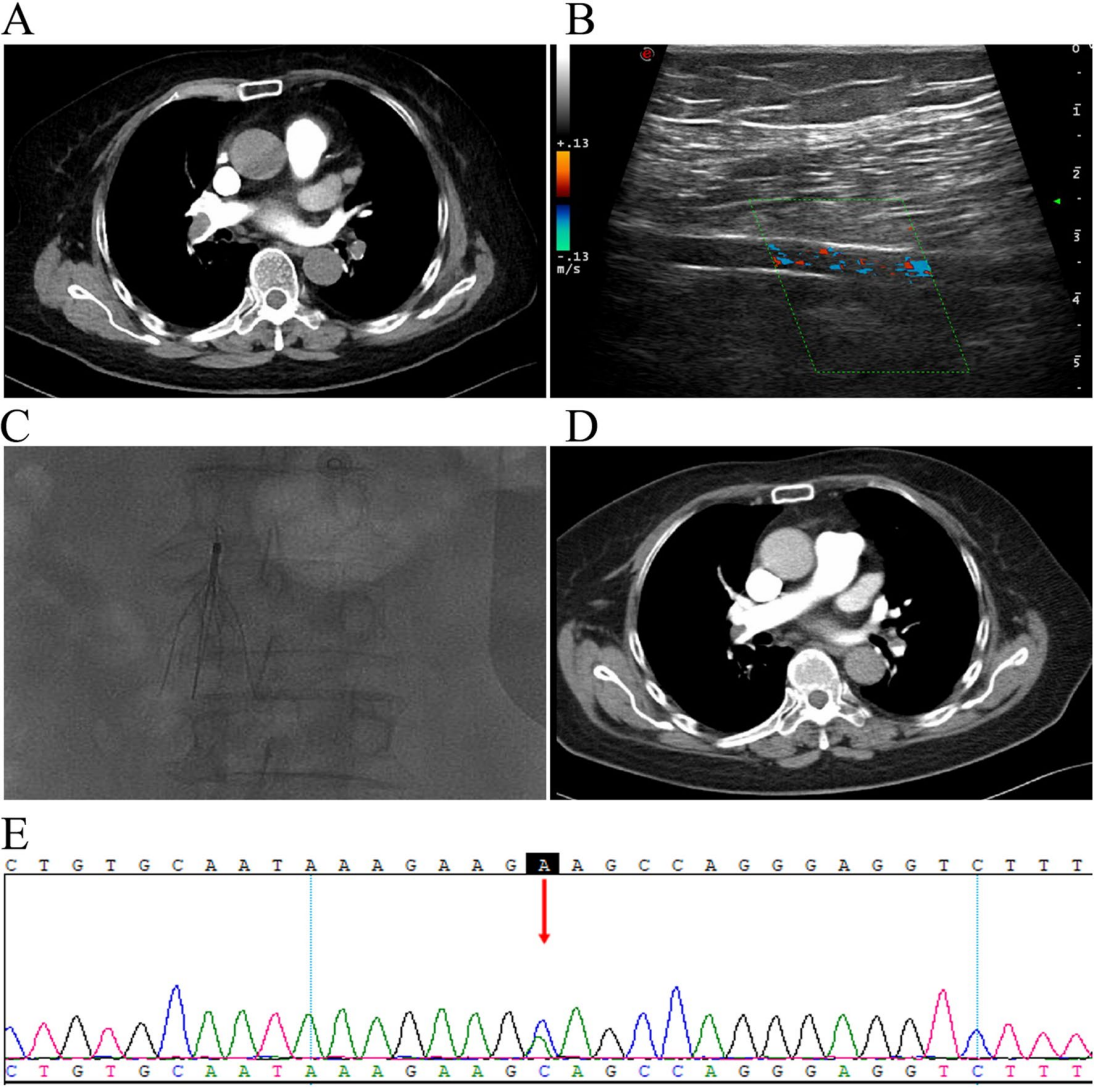
**A**, **B** Computed Tomography Pulmonary Angiography and lower extremity venous ultrasound at initial hospitalization: suggestive of a large filling defect in the right pulmonary artery trunk, consistent with pulmonary embolism. A hypoechoic mass with no blood flow signal was found in the left popliteal vein, suggestive of lower extremity deep vein thrombosis. **C** Placement of filter in inferior vena cava. **D** After 2 weeks of anticoagulant therapy, Computed Tomography Pulmonary Angiography showed a decrease in the size of the main pulmonary artery thrombus. **E** Whole exome sequencing revealed a heterozygous NM000313.3: C200A>C mutation in the PROS1 gene in the patient. Arrows: Sequencing double peaks indicate an A to C mutation

Physical examination revealed no apparent positive signs in the heart and lung examination, including jugular vein distention (JVD) with pulsation, abnormal substernal pulsation, or increased S2. Notably, there was conspicuous edema in the left lower limb. Subsequent routine blood tests, biochemistry, blood gas analysis, thyroid function, and myocardial enzyme spectrum yielded normal results. Coagulation function, however, indicated a significantly elevated d-dimer level at 14,260 µg/L. The electrocardiogram demonstrated sinus bradycardia with a heart rate of 56 beats per minute. Lower extremity ultrasound revealed hypoechoic filling in the left femoral vein and deeper veins, indicative of DVT in the lower extremities. Computed Tomography Pulmonary Angiography (CTPA) confirmed embolism in the right main pulmonary artery (Fig. 1A and B). Urgently, an inferior vena cava filter was implanted (Fig. 1C), and the patient received subcutaneous enoxaparin injections (60 mg every 12 h). Bed rest and regular bowel movements were advised.

Considering the patient’s stable hemodynamics (categorized as intermediate risk) and with full consent from the patient’s family, catheter-directed thrombolysis or aspiration thrombectomy was deemed unnecessary. Upon further medical history inquiry, the patient disclosed an incident occurring 2 days before their visit to the community clinic. Experiencing a sudden loss of consciousness while rising from the bathroom, consciousness was restored approximately two minutes later. With the aid of a nearby wall, the patient regained footing and returned to bed. To avoid undue concern among family members, the patient refrained from discussing this episode until now.

After a 13-day hospitalization, the patient experienced resolution of chest tightness and palpitations, concomitant with a reduction in clot size observed on the CTPA scan (Fig. 1D). Additional blood tests revealed a slight decrease in protein C and protein S activity. However, comprehensive tests, including the anti-nuclear antibody series, anti-neutrophil cytoplasmic antibody, anti-CCP antibody, immunoglobulin and complement, and anti-cardiolipin antibody, returned normal results. Whole exome sequencing was subsequently conducted to explore the patient’s genetic predisposition to thrombophilia. The findings indicated a *PROS1* gene mutation (NM000313.3: C200A>C, C1493-17T>C) (Fig. 1D), a well-documented hotspot mutation associated with protein S deficiency [4], this discovery supported the diagnosis of protein S deficiency (PSD), attributing the patient’s thrombophilia to the identified genetic mutation. Following discharge, the patient was prescribed oral rivaroxaban (15 mg) once daily for lifelong use. A subsequent visit 2 weeks later involved the removal of the inferior vena cava filter. Lower limb ultrasound results demonstrated normalcy in the left femoral vein, thrombus recanalization in the popliteal vein, and unremarkable findings in the right-sided veins, with the patient reporting no significant discomfort.

Over the course of 1 year, monthly telephone follow-ups were conducted. During these follow-ups, the patient consistently reported no recurrence of thromboembolic events. One year later, the patient was admitted for COVID-19, with a normal pulmonary CT scan. After successful treatment, the patient was discharged, marking the conclusion of this complex medical journey.

## Discussion

Looking back at this case, we identify at least two potential pitfalls that could have compromised patient safety and influenced the decision-making process during diagnosis and treatment. The first pitfall is the delayed consideration of pulmonary embolism (PE) in clinical diagnosis, owing to its perceived rarity and nonspecific symptoms. For instance, upon the patient’s initial admission for palpitations and chest tightness, the electrocardiogram indicated sinus tachycardia. Despite this, the treating physician prescribed metoprolol for symptomatic relief without exploring the patient’s lower extremity edema or acquiring a detailed history of bedrest. Subsequent inquiries into the patient’s medical history revealed a brief loss of consciousness during a bathroom visit before experiencing symptoms such as chest tightness and shortness of breath. The patient, experiencing difficulty walking, leaned against the wall to return to bed, with fatigue gradually resolving after 3–5 h. This sequence suggests a potential detachment of a blood clot, resulting in a brief pulmonary embolism and loss of consciousness, followed by possible thrombus self-dissolution or displacement, ultimately leading to the restoration of pulmonary circulation. During this critical juncture, the patient was at a high risk of sudden death, making the subsequent recovery of pulmonary circulation a stroke of luck.

The second pitfall arises from the current understanding that patients with irremovable risk factors, such as genetic abnormalities, necessitate lifelong anticoagulant therapy following survival from PE [3]. Our rationale for initiating genetic testing on the patient was twofold: firstly, the occurrence of fatal DVT and PE within an unusually brief 10-day period of bed rest; and secondly, blood tests revealing slightly diminished levels of protein S and protein C activity. These observations prompted us to explore the patient’s thrombophilia through genetic testing, recognizing its potential decisive impact on shaping the subsequent anticoagulant treatment plan. Without this genetic insight, the patient might have been classified as a sporadic pulmonary embolism case attributed to lower limb pain and immobilization, potentially leading to a standard anticoagulation regimen of 3–6 months. However, such an approach would undeniably heighten the risk of sudden death. Moreover, previous research has demonstrated that protein S deficiency is associated with a tenfold increase in the risk of venous thromboembolism [5]. Furthermore, genetic testing was extended to the patient’s two adult daughters, revealing that each daughter inherited one of the mother’s gene mutations. Although neither daughter had experienced thrombotic events during pregnancy or childbirth, we advised them to adopt necessary preventive measures, such as short-term oral anticoagulant medication during high-risk periods of thrombosis, such as long-distance travel. Their gratitude for this proactive approach was evident.

## Conclusion

In conclusion, this case emphasizes considering PE when presented with non-specific symptoms like palpitations and chest tightness, especially in the presence of unilateral lower limb edema and a history of immobilization. Timely recognition of these signs is crucial for accurate diagnosis. Additionally, our experience highlights the pivotal role of genetic testing for thrombophilia in PE survivors. This proactive approach aids in tailoring an optimal anticoagulant treatment plan, contributing to improved patient outcomes.

## Data Availability

The datasets generated during and/or analyzed during the current study are available from the corresponding author on reasonable request. The materials used in this study are available commercially or can be obtained from the authors upon request.
